# The Human Cytomegalovirus Latency-Associated Gene Product Latency Unique Natural Antigen Regulates Latent Gene Expression

**DOI:** 10.3390/v15091875

**Published:** 2023-09-04

**Authors:** Emma Poole, Jonathan Lau, Ian Groves, Kate Roche, Eain Murphy, Maria Carlan da Silva, Matthew Reeves, John Sinclair

**Affiliations:** 1 Department of Medicine, University of Cambridge, Cambridge CB2 0QQ, UK; jonathan.lau.09@alumni.ucl.ac.uk; 2 Department of Pathology, University of Cambridge, Cambridge CB2 0QQ, UK; 3 Infection Biology, Global Center for Pathogen and Human Health Research, Lerner Research Institute, Cleveland Clinic, Cleveland, OH 44196, USA; grovesi@ccf.org (I.G.); kate@evrysbio.com (K.R.);; 4 University of Sao Paulo, Sao Paulo 05508-060, Brazil; silva.mariac@gmail.com; 5 Infection and Immunity, University College London, London WC1E 6BT, UK; matthew.reeves@ucl.ac.uk

**Keywords:** human cytomegalovirus, latency, chromatin

## Abstract

Human cytomegalovirus (HCMV) infection can lead to either lytic or latent infection, which is dependent on the regulation of the viral major immediate early promoter (MIEP). Suppression of the MIEP is a pre-requisite for latency and is driven by repressive epigenetic modifications at the MIEP during latent infection. However, other viral genes are expressed during latency and this is correlated with activatory epigenetic modifications at latent gene promoters. Yet the molecular basis of the differential regulation of latent and lytic gene expression by epigenetics is unclear. LUNA, a latent viral transcript, has been suggested to be important for HCMV latency and has also been shown to be important for efficient reactivation likely through its known deSUMOylase activity. Intriguingly, we and others have also observed that LUNA enhances latency-associated expression of the viral UL138 gene. Here, we show that in the absence of LUNA, the expression of multiple latency-associated transcripts is reduced during latent infection, which is correlated with a lack of activatory marks at their promoters. Interestingly, we also show that LUNA interacts with the hematopoietic transcription factor GATA-2, which has previously been shown to bind to a number of latency-associated gene promoters, and that this interaction is dependent on the deSUMOylase domain of LUNA. Finally, we show that the deSUMOylase activity of LUNA is required for the establishment and/or maintenance of an open chromatin configuration around latency-associated gene promoters. As such, LUNA plays a key role in efficient latency-associated viral gene expression and carriage of viral genome during latent carriage.

## 1. Introduction

Between 60 and 99% of global populations carry HCMV, depending on demographics; in part, this prevalence is made possible by the ability of the virus to establish lifelong persistence in the infected host. After primary lytic infection, HCMV can establish a quiescent or latent infection, which helps it avoid immune clearance [1]. The lytic (productive) lifecycle comprises a temporal cascade of immediate early (IE), early, and late gene expression and results in the generation of progeny virions. During lytic infection, the major immediate early promoter (MIEP), which drives the expression of key lytic cycle genes (IE genes), is associated with activatory markers of transcription. In contrast, during the latent lifecycle, when there are no infectious virions produced, the MIEP is associated with repressive chromatin marks [2]. Although it is likely that a number of sites of latency may exist in vivo [3,4,5], one defined site of HCMV latency is in undifferentiated cells of the myeloid lineage such as CD34+ progenitor cells and their CD14+ monocyte derivatives [2]. However, upon their differentiation into terminally differentiated myeloid cells such as macrophages or dendritic cells, the MIEP becomes transcriptionally active, driving the lytic gene expression cascade and resulting in the production of virions [2].

During latent infection of undifferentiated myeloid cells, the MIEP is known to be targeted for repression to suppress the expression of key lytic IE genes by an overall balance of MIEP repressors [6,7,8]. However, it is clear that, concomitantly with repression of the MIEP, promoters of latency-associated genes must be activated. Although the extent of the latency-associated transcriptome is far from clear, expression and latency-associated functions of a number of viral genes have been identified during latent infection, and these include US28, UL138, and viral IL-10 (vIL-10) [9,10,11,12,13,14,15,16,17,18]. However, what mediates the promoter activity of these genes during latent infection has not been well addressed beyond demonstration that the promoters are associated with active chromatin marks, although the UL133-UL138 locus has been extensively studied during both lytic and latent infection [19].

Another viral gene that has been shown to be expressed during latency is LUNA. It was one of the first genes identified to be transcribed during natural latency [20] and has been shown to be important for efficient reactivation [21,22]—key to this appears to be LUNA’s deSUMOylase activity [22]. We have also previously shown that LUNA expression is driven, at least in part, by the myeloid transcription factor GATA-2 [23]. GATA-2 is known to drive the expression of a number of myeloid-specific genes, which is consistent with the ability of myeloid cells to support the LUNA gene expressed in an IE-independent manner [23,24]. In defining a key role for LUNA in reactivation, we observed that deletion of LUNA had a reproducible impact on UL138 gene expression in CD34+ cells [22], with a similar observation also reported in CD14+ monocytes [20].

In this manuscript, we further investigate this transcriptional phenotype and demonstrate that LUNA is important for the expression of multiple latency-associated transcripts, which correlates with increased levels of activatory epigenetic marks at latent promoters. In silico analyses reveal that multiple latent promoters encode binding sites for the hematopoietic transcription factor GATA-2, and interestingly, we show that LUNA interacts with transfected GATA-2 by co-immunoprecipitation (Co-IP). Finally, we link GATA-2 binding and the formation of an open chromatin conformation at latent viral promoters with the deSUMOylase motif of the LUNA protein. Together, these data argue that LUNA contributes to the regulation of expression of latency-associated HCMV gene expression in myeloid cells through an interaction with an important hematopoietic transcription factor, GATA-2.

## 2. Materials and Methods

### 2.1. Cells and Viruses

THP1 cells were cultured in RPMI-1640 (Gibco) supplemented with 10% FCS and 5% penicillin/streptomycin in 5% CO_2_ at 37 degrees. CD14+ cells were isolated from venous blood as described previously [25,26]. TB40E-SV40GFP and TB40E-IE2YFP viruses have been described previously [7].

TB40E-SV40-GFP BAC was used to generate the LUNA mutant, LUNA knockout, and revertant viruses. For this, gBLOCKs from IDT (Coralville, IA, USA) were utilised with primers for recombineering using the GalK/2-DOG selection method as described previously [27], using the following primers: 5′ GalK insert LUNA (primer).

TACCGCTTCGACGTCTTTGTCCGGTCAGGATCAGTGCCCGGGACAGTCCGCCTGTTGACAATTAATCATCGGCA 3′ GalK insert LUNA (primer) GGTCTCTTTCCACGGAGCAACGTCATGCGCGGCGCCGTCTCCGAGTTTCTTCAGCACTGTCCTGCTCCTT LUNA G81A stop (gBLOCK) TACCGCTTCGACGTCTTTGTCCGGTCAGGATCAGTGCCCGGGACAGTCCGGCTTGAGTGTCCGAGTCCTCGTCGCCGCTGGCCTCCTCGAAGCCGGCAAACATGGCTTCGGACAGGGGGGTCGGCGTCGGTGTGGATGAGAGGTCATCTTCGTCGTCCTCTTCCTCTTCTTCCTCCTCTTCCTCGGTGGGTGGTAATCCGGGGGACTGCGGGAGAAACTCGGAGACGGCGCCGCGCATGACGTTGCTCCGTGGAAAGAGACC LUNA G233C (gBLOCK)TACCGCTTCGACGTCTTTGTCCGGTCAGGATCAGTGCCCGGGACAGTCCGGCTTGGGTGTCCGAGTCCTCGTCGCCGCTGGCCTCCTCGAAGCCGGCAAACATGGCTTCGGACAGGGGGGTCGGCGTCGGTGTGGATGAGAGGTCATCTTCGTCGTCCTCTTCCTCTTCTTCCTCCTCTTCCTCGGTGGGTGGTAATCCGGGGGACTCCGGGAGAAACTCGGAGACGGCGCCGCGCATGACGTTGCTCCGTGGAAAGAGACC.

LUNA C234G (gBLOCK) TACCGCTTCGACGTCTTTGTCCGGTCAGGATCAGTGCCCGGGACAGTCCGGCTTGGGTGTCCGAGTCCTCGTCGCCGCTGGCCTCCTCGAAGCCGGCAAACATGGCTTCGGACAGGGGGGTCGGCGTCGGTGTGGATGAGAGGTCATCTTCGTCGTCCTCTTCCTCTTCTTCCTCCTCTTCCTCGGTGGGTGGTAATCCGGGGGACTGGGGGAGAAACTCGGAGACGGCGCCGCGCATGACGTTGCTCCGTGGAAAGAGACC.

5′ LUNA seq (60.4) (5′ primer for sequencing the mutants) GCG TGT TGC ACG CTC ACC 3′ LUNA seq (59.8) (3′ primer for sequencing the mutants) CCG CCG TGG GTT TTG GAC 5′ amp LUNA gB (55) TACCGCTTCGACGTCTTTG.

3′ amp LUNA gB (55.5) GGTCTCTTTCCACGGAGC.

### 2.2. Chromatin Immunoprecipitation

Chromatin immunoprecipitations were carried out as described previously [7]. After sonication, DNA was immunoprecipitated with antibodies specific for H3K4me3 or H3K9me3 (Santa Cruz) at 1:100 dilution, as indicated, and the promoter regions for each gene were amplified by PCR with the following forward and reverse primers: LUNA promoter GCGGGTTCCAATCAGCAGCAGC and CAGCTACCTTGGCACCTCCGG. UL138 promoter CGGGGTACCCGGCGTAAGAGAAACCGA and CCGCTCGAGGCCAACTGTCCTGGTGGT TLR4 AAGCCGAAAGGTGATTGTTG and CTGAGCAGGGTCTTCTCCAC ZNF180 TGATGCACAATAAGTCGAGC and TGCAGTCAATGTGGGAAGTC. DNA was analysed using quantitative PCR using QuantiTect SYBR^®^ Green RT-qPCR kit according to the manufacturer’s instructions and the samples were amplified and detected using an ABI 7500 Fast Real-Time PCR machine (95 °C for 15 s and 60 °C for 45 s), as described previously [7].

### 2.3. Immunoprecipitation (IP)

Immunoprecipitation analysis was carried out as described previously [28] using anti-Flag antibody at 1:100 dilution (Thermo Fisher) or the matched isotype control (Abcam). The IP samples were analysed by Western blot using a GATA-2-specific antibody at 1 in 1000 dilution (Cell Signalling).

### 2.4. Drug Treatments

Isopeptidase inhibitor, G5 (Sigma), was used as described previously [22]. First, 1 uM of G5 was added to the cells 30 min post-transfection with plasmids (expressing GATA-2 or flag-tagged LUNA), and then cells were harvested for immunoprecipitation 48 h post-transfection.

### 2.5. qRT-PCR

Quantification of viral and cellular mRNAs was carried out by SYBR green detection using primers described previously [7], except vIL-10 was detected using the following primers: Forward 5′-AAA ACC TAC GTT GCA ACG TGA GGA-3′ Reverse 5′-CAA CCT AAC AGA GGG CAT TGC-3′.

### 2.6. Droplet PCR

Genome copy number was determined using droplet digital PCR as described previously [29,30]. Briefly, cells were harvested for DNA purification (Qiagen Blood Mini) and then the viral genome was quantified using QX200 droplet digital PCR (Biorad) with primers and probes specific to viral gB (Primer Design) and cellular RPP30 (Biorad).

## 3. Results

### 3.1. LUNA Expression Promotes an Open Chromatin Conformation at Latent Promoters and Increased Latent Viral Gene Expression

**Figure 1:**
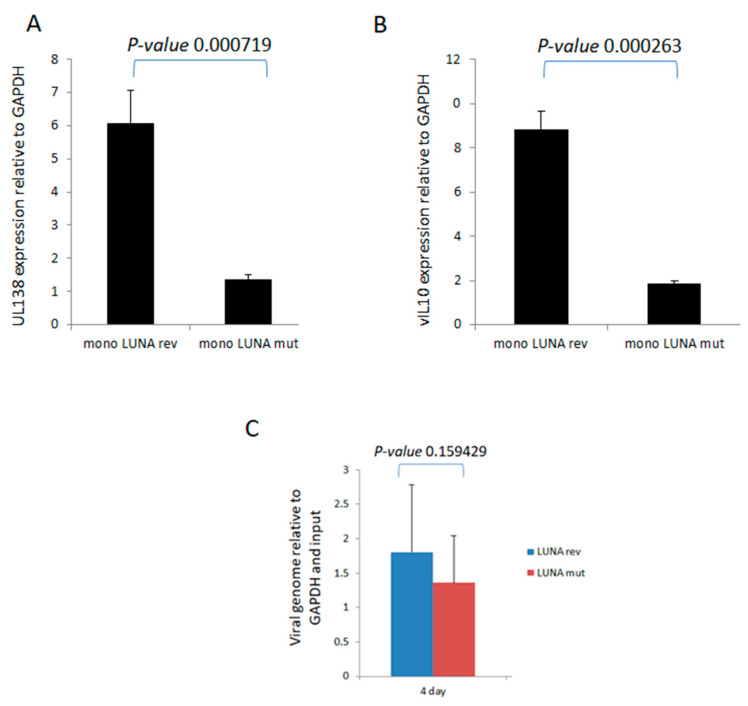
LUNA is required for optimal expression of latency-associated genes UL138 and vIL-10 and genome carriage in CD14+ monocytes. CD14+ monocytes were infected with TB40E-LUNA-Revertant (mono LUNA rev) or TB40E-LUNA-Deletion virus (mono LUNA mut) and then latency was established for 4 days prior to harvesting the RNA for RTqPCR analysis of the latency-associated mRNA UL138 (**A**), viral IL10 (vIL10) (**B**), and GAPDH housekeeping gene. Additionally, the same infections were analysed for viral genome carriage at 4 dpi (**C**). Graphs show representative triplicate samples from duplicate experiments. Standard deviation about the mean and *p*-values of significance from Student’s *t*-test are shown.

To investigate directly whether LUNA is important for latency-associated viral gene expression, we analysed the levels of UL138 and vIL-10 RNA during latent infection in the absence of LUNA. Recombinant viruses were generated in which a premature stop codon was engineered at the start of LUNA, resulting in viruses unable to express LUNA protein but which did not disrupt the UL82 gene on the complementary DNA strand (TB40E-LUNAmut), which was then controlled for through production of a revertant virus with LUNA repaired (TB40E-LUNArev). It is worth pointing out that this equivalent mutation of LUNA in the context of the Merlin clinical isolate of HCMV had little impact on the growth of Merlin in fibroblasts and the expression of viral gene products, including UL82 [22]. The data show that in the absence of LUNA, there was a decrease in expression of both UL138 (Figure 1A) and vIL-10 (Figure 1B) in latently infected monocytes. Importantly, these decreases in expression of UL138 and vIL-10 could not be explained by differences in viral genome copy number, as at the time of analysis, we observed that genome carriage was similar in cells infected with each virus (Figure 1C).

**Figure 2:**
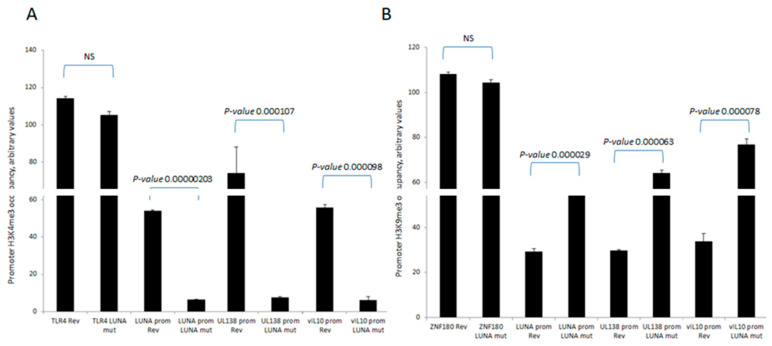
LUNA is required for the activation of latency-associated promoters UL138, LUNA, and vIL-10. CD14 monocytes were infected with TB40E-LUNA-Revertant (rev) or TB40E-LUNA-Deletion virus (LUNA mut) for 4 days before harvesting for ChIP analysis. Viral promoters for vIL-10 (vIL-10 prom), LUNA (LUNA prom), and UL138 (UL138 prom) were tested for the presence of activatory histone mark H3K4me3 alongside cellular TLR4 as a positive control (**A**). The same viral promoters were tested for the presence of the repressive histone mark H3K9me3 alongside cellular ZNF180 as a positive control (**B**). Graphs show representative triplicate samples from duplicate experiments. Standard deviation about the mean and *p*-values of significance from Student’s *t*-test are shown.

To further understand the mechanisms important for these differences in levels of UL138 and vIL-10 gene expression in the presence or absence of LUNA, we analysed the chromatin structure around the promoters of these latency-associated genes (Figure 2). Consistent with the expression data for UL138 and vIL-10, we saw evidence of H3-K4 methylation—a marker of recent transcription activity—on the promoters of these latency-associated genes in cells infected with wild-type HCMV (Figure 2A). In contrast, there was a clear decrease in H3-K4 methylation at the UL138, LUNA, and vIL-10 promoters upon infection with a LUNA deletion virus (Figure 2A) and a concomitant increase in repressive histone H3-K9 methylation marks (Figure 2B). Taken together, these data argued that the regulatory impact of LUNA on latent gene expression was manifest through epigenetic changes occurring at latency-associated promoters.

### 3.2. LUNA deSUMOylase Motif Is Important for Binding to GATA-2 and Promoting Changes in Chromatin Structure

**Figure 3:**
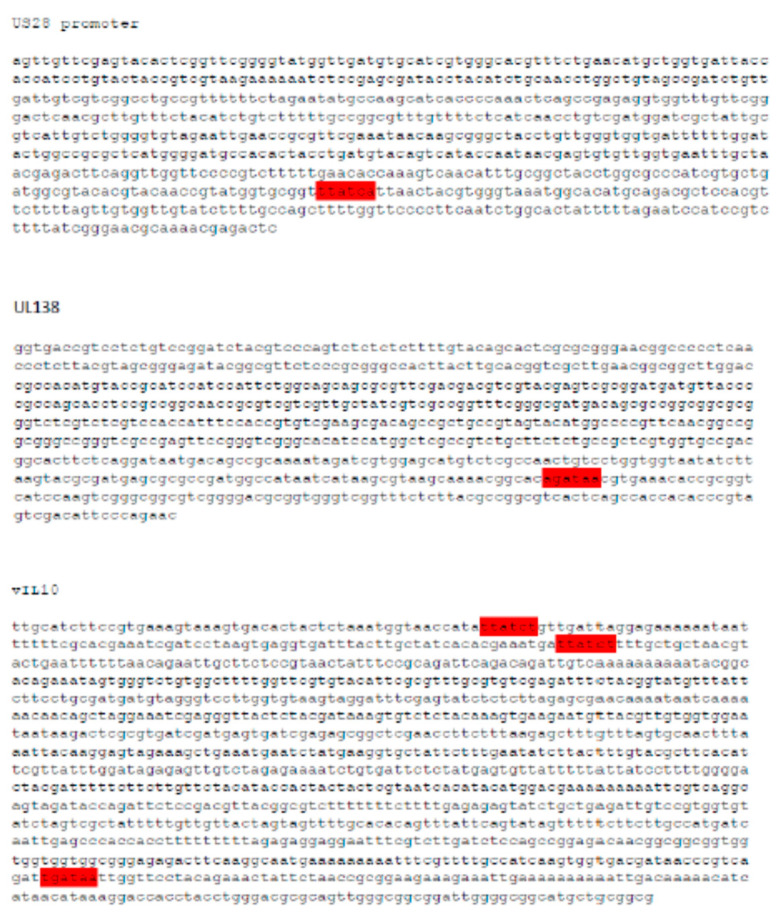
Latency-associated promoters contain GATA-2-binding sites. The sequences for the promoter regions of viral latency-associated genes vIL-10, US28, and UL138 were analysed for the presence of GATA-2-binding sites (highlighted in red).

We previously identified that the hematopoietic transcription factor GATA-2, which plays an important role in host gene expression in myeloid cells, is also important for the expression of both LUNA and UL144 mRNAs during latency by GATA-2 binding to their promoters [23]. Intriguingly, an in silico analysis of additional latency-associated gene promoters clearly identified potential GATA-2-binding sites in the UL138, vIL10, and US28 promoters, suggesting that GATA-2 protein activity could be pivotal for the expression of multiple latent HCMV transcripts, providing a general mechanism for the regulation of latent gene expression (Figure 3).

**Figure 4:**
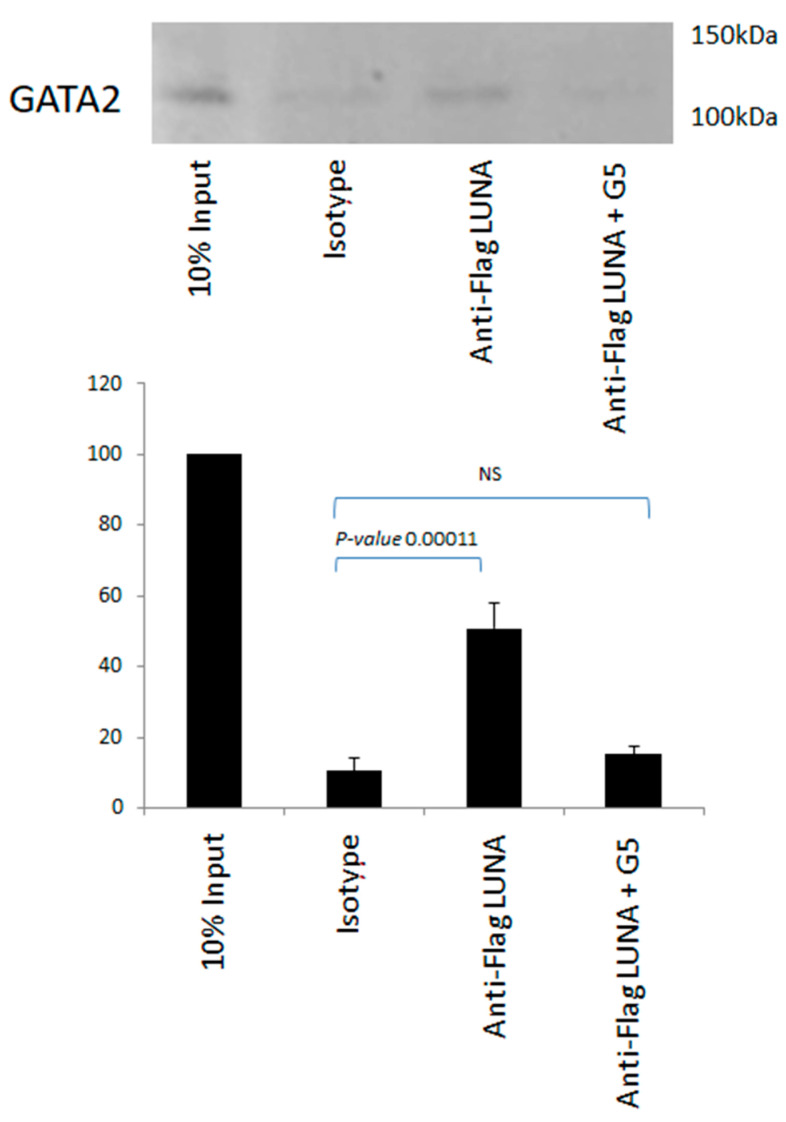
The interaction of LUNA with GATA-2 is blocked by an isopeptidase inhibitor. THP1 cells were electroporated with GATA-2 and flag-tagged LUNA in the presence and absence of isopeptidase inhibitor G5. Cells were then immunoprecipitated with anti-Flag or an isotype control and Western-blotted for GATA-2. Densitometry was carried out on two repeat experiments run in duplicate, and data represent standard deviations about the mean with error bars from Student’s *t*-test.

There is no evidence to suggest that LUNA can function as a general transcription factor; thus, we hypothesised that LUNA could regulate latent gene expression via interaction with GATA-2. To investigate whether LUNA might enhance expression from GATA-2-bearing promoters by a direct interaction between LUNA and GATA-2, we carried out interaction assays using co-immunoprecipitation in cells overexpressing GATA-2 and LUNA, which showed a clear interaction between GATA-2 and LUNA (Figure 4). Furthermore, this interaction could be reduced in the presence of G5—an isopeptidase inhibitor that binds to LUNA and blocks its function (Figure 4). These data argue that LUNA and GATA-2 are able to interact with each other and that this interaction is dependent on the deSUMOylase motif of LUNA.

**Figure 5:**
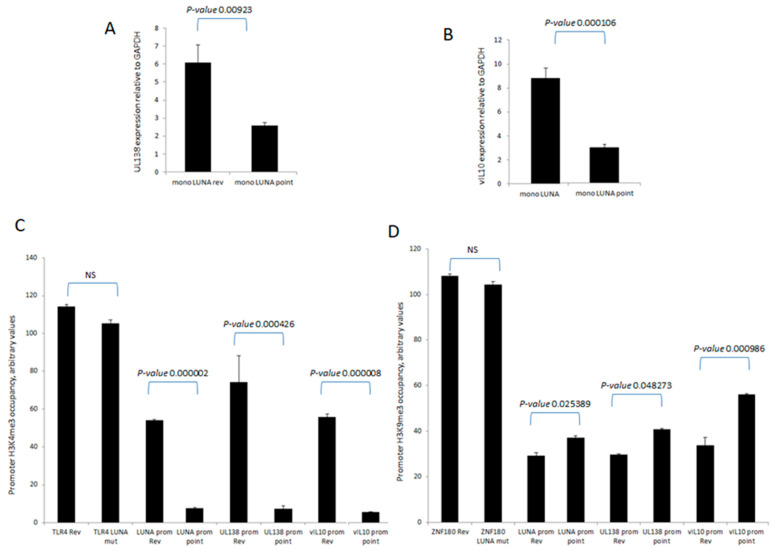
LUNA deSUMOylase function is required for the activation of latency-associated promoters UL138, LUNA, and vIL-10. CD14 monocytes were infected with TB40E-LUNA-Revertant (rev) or deSUMOylase-deficient point mutant virus TB40E-LUNA-point (LUNA point) for 4 days before either RT-qPCR for vIL-10 (**A**) or UL138 (**B**) or harvesting for ChIP analysis. Viral promoters for vIL-10 (vIL10 prom), LUNA (LUNA prom), and UL138 (UL138 prom) were tested for the presence of activatory histone mark H3K4me3 alongside cellular TLR4 as a positive control (**C**). The same viral promoters were tested for the presence of the repressive histone mark H3K9me3 alongside cellular ZNF180 as a positive control (**D**). Graphs show representative triplicate samples from duplicate experiments. Standard deviation about the mean and *p*-values of significance from Student’s *t*-test are shown.

We observed that LUNA expression resulted in the formation of active chromatin on viral latency-associated promoters, that all these promoters contained GATA-2 transcription factor binding sites, and that LUNA interacted with GATA-2 in a deSUMOylase-dependent manner. Thus, we hypothesised that the chromatin signature observed with a LUNA deletion virus should be phenocopied with a LUNA deSUMOylase mutant. To test this, we generated a LUNA mutant virus in which we introduced a point mutation in the deSUMOylase domain of LUNA, rendering LUNA devoid of deSUMOylase activity (TB40E-LUNApoint), and tested whether the loss of the deSUMOylase activity from LUNA affected the transcription of viral genes. An analysis of infected cells shows that the expression of both vIL-10 and UL138 is reduced in cells latently infected with the TB40E LUNApoint compared to the control (Figure 5A,B). Next, we conducted a ChIP analysis of multiple latency-associated promoters to detect H3-K4me and H3-K9me modification after latent infection with wild-type and LUNA point mutant viruses. As the data show, elevated H3-K9me and reduced H3-K4me were clearly evident on latent promoters in mutant-virus-infected cells (Figure 5C,D). Therefore, our data suggest that the regulation of viral transcription and chromatin structure during latency by LUNA requires a functional deSUMOylation motif.

### 3.3. LUNA Expression Contributes to an Extended Lifespan in Infected CD14+ Monocytes

**Figure 6:**
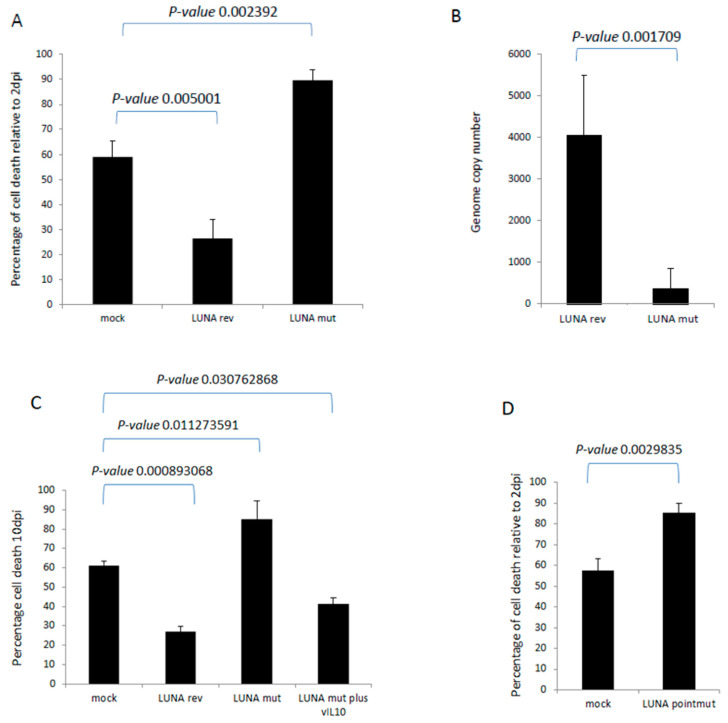
LUNA increases cell survival during latency in CD14+ monocytes. CD14+ monocytes were infected with either TB40E-SV40GFP-LUNA mutant virus (LUNA mut) or TB40E-SV40GFP-LUNA revertant virus (LUNA rev) and assayed for cell death relative to cell death 2 dpi (**A**). Alternatively, cells were harvested for DNA analysis by droplet PCR (**B**). The same cells and viruses were used to infect cells at equivalent MOIs for 10 days, as for A and B, but in addition to recombinant viral IL-10 (vIL-10), where indicated, and cell death was measured by trypan blue staining (**C**). Finally, the same experiment (**A**) was repeated using the TB40E LUNApointmut virus (**D**). Graphs show representative triplicate samples from duplicate experiments. Standard deviation about the mean and *p*-values of significance from Student’s *t*-test are shown.

Finally, we wished to determine whether these LUNA-mediated changes could have any impact on the biology of latency. It is well established that HCMV infection extends the lifespan of classically short-lived CD14+ monocytes, which could be important for virus dissemination in vivo. Furthermore, we have observed that the expression of vIL-10 enhances the survival of infected CD34+ cells in long-term culture. Thus, we hypothesised that the defect in latency-associated gene expression observed in the absence of LUNA could have an impact on monocyte survival. To this end, CD14+ cells were infected with wild-type or a LUNA deletion virus and cultured for 10 days, and monocyte viability was assessed. The data show that viral infection improves cell survival compared to mock cells (Figure 6A), consistent with previous studies [14]. In contrast, cell viability was decreased in cells infected with the LUNA deletion virus (Figure 6A). This change in viability was mirrored by genome carriage, whereby infection with the LUNA deletion virus also led to a significant decrease in viral DNA by ddPCR (Figure 6B). Our original hypothesis proposed that the loss of vIL-10 was likely responsible for this phenotype and, consistent with this, the drop in cell viability could be reversed by the addition of recombinant vIL-10 (Figure 6C).

## 4. Discussion

Previous studies have demonstrated a clear role for the latency-associated gene product, LUNA, in latent carriage and reactivation from latency [21,22]. Here, we detail how LUNA is required for latent infection.

Whilst the absence of LUNA does not prevent the establishment of latency in CD14+ monocytes, we show here that it significantly enhances the expression of latency-associated genes, which we hypothesise is at least in part via the myeloid transcription factor GATA-2; this included the expression of vIL-10, which was significantly reduced in the absence of LUNA.

We have previously shown that vIL-10 drives the expression of cellular IL-10 in monocytes [13,31], and others have shown that vIL-10 can prevent inflammatory cytokine production, which likely aids monocyte survival [32]. Additionally, we know that cellular IL-10, induced by vIL-10, is pro-survival in an anti-FAS mediated signalling mechanism via PEA-15 in myeloid progenitors [12,14]. In essence, latency-associated expression of vIL-10 during latency would be predicted to enhance the survival of long-term latently infected cells. Fully consistent with this, we observed reduced monocyte survival during latent infection in the absence of LUNA (and the resulting reduction in vIL-10 expression), which could be rescued by the addition of exogenous/recombinant vIL-10. Interestingly, our previous analysis in CD34+ cells with the Merlin strain of HCMV did not demonstrate a decrease in genome carriage in the absence of LUNA at later time points, as shown for TB40E in CD14+ cells in this study (Figure 6); this may be due to the different strain of virus and the different cell type used.

It is important to highlight that vIL-10 has pleiotropic roles in HCMV infection, with key roles in immune regulation. Thus, the enhancement of vIL-10 production by LUNA during latency could also be important for immune evasion during latency in vivo. For example, vIL-10 directly modulates the immune response [33] and the vIL-10-induced cellular IL-10 has been shown to contribute to the immune evasion microenvironment [34,35]. Additionally, vIL-10 mediates some aspects of cellular IL-10 signalling directly [36].

Our previous work has shown that LUNA is a functional deSUMOylase [22], and this function of LUNA causes disruption of PML bodies during latency, likely to aid reactivation [22]. Interestingly, we now find that the deSUMOylase function of LUNA is also important for the regulation of latency-associated gene expression. Whilst it is likely that latency-associated disruption of PML bodies may aid transcription from latency-associated gene promoters, our data also suggest that the function of LUNA during latency also involves an interaction with the myeloid transcription factor GATA-2. GATA-2 transcription factor binding sites reside in a number of the latency-associated gene promoters we have analysed, and our observation that LUNA binds to GATA-2 (via the deSUMOylation motif) suggests that activation of latency-associated gene expression by LUNA is modulated by crosstalk between LUNA and GATA-2. The activity of GATA-2, as well as co-factors such as Friend of GATA (FOG) [37,38], is regulated by changes in sumoylation status. Specifically, sumoylation attenuates their transcriptional activity, which would provide a model for how a deSUMOylase (whether host- or viral-derived) could increase GATA-2 activity. In future studies, it may be informative to generate a virus that expresses GATA-2 in the absence of LUNA, which should rescue the phenotypes observed with the LUNA kO viruses. Indeed, host-derived deSUMOylases promote GATA-2 de-sumoylation [39]—which will be important for general transcription in hematopoietic cells—and LUNA likely enhances this phenotype by increasing the levels of deSUMOylated GATA-2 in a latent cell. This model would explain why latent gene expression is seen in LUNA mutant-virus-infected cells but is enhanced in cells expressing LUNA.

PML is generally repressive for viral IE gene expression, which would be advantageous during latency to help repress lytic gene expression. However, it is at present unclear how the virus maintains a repressive chromatin structure around the MIEP whilst at the same time allowing expression of latency-associated genes. The dispersal of repressive PML bodies during latency would be predicted to be disadvantageous for maintaining the general repressive chromatinization of the viral genome observed during HCMV latency; PML disruption is known to lead to activation of HCMV IE gene expression and gene expression of other viruses [40,41]. For instance, latent infection with HSV-1 does not lead to the disruption of PML bodies; this only occurs as the lytic cascade is initiated with the expression of ICP0. Similarly, PML bodies are not disrupted during EBV latency until lytic infection is initiated via the BNRF1 protein. However, it is also known that PML bodies are not required for long-term repression of the MIEP during HCMV latency [42] but rather MIEP repression is maintained in undifferentiated myeloid cell types, at the chromatin level, due to a differentiation-specific surfeit of cellular MIEP repressors [2], as well as an absence of MIEP activators such as viral pp71 [43]. It also cannot be ruled out that there are other repressive factors, perhaps yet unidentified ND10-independent factors, that are present during latency.

In contrast, reactivation is likely regulated by the increase in differentiation-dependent transcriptional activators of the MIEP, coupled with a decrease in MIEP repressive factors, which can be enhanced by inflammatory signals that then allow viral IE gene expression [44] and progression of the lytic cascade [45,46,47,48].

Taken together, we believe that the deSUMOylation function of LUNA not only ensures that reactivation from latency is primed to go, by removing repressive PML bodies in latently infected cells, but is also crucial for latent carriage by enhancing the transcription of latency-associated genes.

## Data Availability

All new data is presented in the manuscript.
